# Investigation of taurine and aqueous garlic extract diet supplementation effect on the healing of rat osteoporotic fractures

**DOI:** 10.55730/1300-0144.5555

**Published:** 2023-02-02

**Authors:** Mesut MISIRLIOĞLU, İzzet BİNGÖL, Coşkun GENÇ, Aylin AKBULUT, Mert OCAK, Neziha Yağmur DIKER, Ahmet ÖZMERİÇ, Gökhan KOCA, Melike BAHÇECİTAPAR, Mehmet ŞENES, Ayşegül FIRAT, Fevziye Figen KAYMAZ, Hakan Hamdi ÇELİK, İffet İrem ÇANKAYA, Meliha KORKMAZ

**Affiliations:** 1 Department of Orthopedics and Traumatology, University of Health Sciences Ankara Oncology Education and Training Hospital, Ankara, Turkey; 2 Department of Nuclear Medicine, University of Health Sciences Ankara Training and Research Hospital, Ankara, Turkey; 3 Department of Anatomy, Faculty of Dentistry, Ankara University, Ankara, Turkey; 4 Department of Pharmaceutical Botany, Faculty of Pharmacy, Hacettepe University, Ankara, Turkey; 5 Department of Orthopedics and Traumatology, University of Health Sciences Ankara Training and Research Hospital, Ankara, Turkey; 6 Department of Statistics, Faculty of Science, Hacettepe University, Ankara, Turkey; 7 Department of Medical Biochemistry, University of Health Sciences Ankara Training and Research Hospital, Ankara, Turkey; 8 Department of Anatomy, Faculty of Medicine, Hacettepe University, Ankara, Turkey; 9 Department of Histology and Embryology, Faculty of Medicine, Hacettepe University, Ankara, Turkey; 10 Department of Anatomy, Faculty of Medicine, Hacettepe University, Ankara, Turkey

**Keywords:** Taurine, aqueous garlic extract, osteoporosis, ovariectomized rat, femur fracture

## Abstract

**Background/aim:**

We have evaluated the effects of taurine and aqueous garlic extract (AGE) as a dietary supplement on osteoporotic fracture (OPF) healing in the ovariectomized rat femur fracture model.

**Materials and methods:**

In this experimental animal study, twenty-four osteoporosis-remodeled female Wistar albino rats were randomly divided into 3 groups (n: 8) according to their supplemented diet; control, taurine, and AGE groups. Unilateral femur middiaphysis mini-open osteotomy was stabilized with Kirschner wires. Six weeks after osteotomy, the rats were sacrificed before the femurs were harvested and OPF healing was evaluated with biochemical, histologic, microcomputed-tomography, and scintigraphic methods.

**Results:**

As an indicator of the antiosteoporotic effect, the calcium levels of the taurine group were significantly lower than the AGE and control groups in biochemical analyzes (p < 0.01). In histological studies, the new bone diameter and new bone volume values of the taurine group were significantly higher than the control group (p = 0.002 and p = 0.032, respectively), while higher trabecular-compact callus was observed in the taurine and AGE groups, respectively, compared to the control group. In morphological analyses, taurine and AGE groups had significantly higher bone volume/tissue volume, trabecular number, bone surface density, and lower trabecular separation than the control group (p < 0.05). The scintigraphic imaging showed a significant increase in osteoblastic activity of the taurine group compared to the control group (p = 0.005).

**Conclusion:**

Taurine and AGE have positive anabolic effects, respectively, on the healing of OPFs, demonstrated by biochemical, histological, morphological, and scintigraphic methods.

## 1. Introduction

Osteoporosis is a common metabolic bone disease, closely related to diet and caused by an imbalance of bone formation and resorption. It is characterized by progressive bone mass loss, bone microarchitecture deterioration, and increased fragility [1,2]. Osteoporotic fractures (OPF), which are an important health problem due to decreased bone healing capacity, morbidity, and mortality, are frequently seen in postmenopausal elderly women [**3].** Recently, the interest in the consumption of natural alternative agents and herbs has been rising due to the unsatisfactory results, side effects, related complications of long-term treatment, and high costs of currently available conventional osteoporosis treatment modalities [4–7]. Taurine and garlic have been used in the treatment of osteoporosis for years, as they are easily available without a prescription, cost-effective with minimal side effects even used for a long time [4,5].

Taurine (2-aminoethanesulfonic acid) is a semiessential amino acid that has beneficial effects on hypertension, congestive heart failure, diabetes, convulsion, obesity, and cancer. It can be used as an additive to baby milk formula, eye-ear drops, and antiaging agents [8–10]. In addition to its benefits in improving skin and bone health, it is accepted that its deficiency causes growth retardation, deficiency of tissue differentiation, and immune development [11]. Studies have shown that taurine is abundant in the bone matrix, and it increases endurance performance [10], matrix mineralization, osteoblast differentiation, and bone tissue formation. It reduces bone loss by inhibiting osteoclastogenesis [11,12]. Animal experiments have supported that taurine supplementation reduces alveolar bone resorption [8], prevents bone loss in ovariectomized rats [6,11,13], and has anabolic effects on bone health in growing rats [9,12], without significant side effects or toxicity. Oral taurine tablets have been reported to reduce postoperative oxidative stress in elderly patients with hip fractures [14]. Although the positive effects of taurine on growth and bone metabolism have been determined, its effect on OPF healing remains unknown.

Garlic (Allium sativum) is a safe, easily accessible plant that has long been used as seasonings for cooking, food additive, and herbal agent in traditional medicine [7,15]. Previous studies have shown that garlic has several biological functions, including antibacterial, antiviral, antifungal, antioxidant, antiinflammatory, antithrombotic, anticholesterol, antidiabetic, antineoplastic, antiobesity, antiaging, antiapoptotic, immunomodulatory, cardioprotective, radioprotective, and hepatoprotective activities [5,15–19]. Furthermore, with its antiosteoporotic effects, garlic is a phytoestrogen, that reduces the risk of postmenopausal fractures [2,7]. It has been shown that garlic significantly protects against hypogonadal bone loss in ovariectomized rats, with its estrogen-like antioxidant and antiinflammatory effects [19], and reduces bone loss in postmenopausal osteoporotic women [20]. It has been reported that aqueous garlic extract (AGE) is effective in the treatment of periodontal bone diseases by increasing bone marrow cellularity and has antioxidant effects in postmenopausal osteoporosis [16], and improves bone density by inhibiting osteoclastogenesis, increasing bone turnover, and increasing calcium absorption from the intestines [5]. Although many beneficial effects of AGE on bone metabolism and also in the treatment of osteoporosis have been demonstrated [5,20], its effects on bone fracture have not been studied yet.

The positive effects of taurine and AGE on bone metabolism have been known for a long time and both agents are used in the treatment of osteoporosis. Regarding that, we hypothesized that taurine and AGE could be effective candidate agents for enhancing OPF healing. To the best of our knowledge, this study is the first study in the literature investigating the effects of taurine and aqueous garlic extract on an ovariectomy-induced experimental osteoporotic bone fracture model. This study aimed to evaluate the effects of taurine and AGE on OPF healing by biochemical, histomorphologic, and scintigraphic methods.

## 2. Materials and methods

### 2.1. Preparation of taurine and AGE

The taurine powder (Farmatek Chemical, İstanbul/Turkey-TR-34-K062203) has been diluted at 3 g per scale with saline before orogastric administration [9]. To avoid the strong and irritating odor of raw garlic and feeding difficulties, and to optimize and standardize the dose given, we preferred to administer freshly prepared AGE to rats by the orogastric route [8,21]. The garlic bulbs, purchased from the local market, were peeled, blended, and soaked in distilled water for 12 h. The mixture of crushed garlic and distilled water was stirred and filtered through filter paper. The solvent was removed by evaporation under reduced pressure to obtain dry aqueous extracts. Then extract was stored in an airtight container at 4 °C [21]. The garlic extract was prepared at the Department of Pharmacognosy (Hacettepe University Faculty of Pharmacy).

### 2.2. Animals and study design

All the experiments were performed between May and November 2020, at Ankara Training and Research Hospital Hüsnü Sakal Experimental and Clinical Research Center (Ankara, Turkey) with the approval of the Local Ethics Committee (19.03.2020/610). Twenty-four Wistar albino female rats, 16 weeks old, weighing 300–325 g, were placed individually in cages and maintained at a 12-h light-dark cycle at 24 ± 2 °C, 60 ± 5% humidity, with ad libitum access to deionized water and standard laboratory phytoestrogen-free rat chow diets (Bil-Yem Co, Ankara, Turkey). After a 1-week adaptation period, bilateral ovariectomy was performed (by G Koca) under anesthesia to induce an estrogen-deficient postmenopausal osteoporosis model. Twelve weeks after ovariectomy, osteoporotic rats underwent femoral osteotomy and were randomly divided into 3 equal groups (n = 8) according to the food supplement; taurine, AGE, and control groups. For 6 weeks from the first day after osteotomy, 2 mL of taurine at a dose of 500 mg/kg/day [9] by oral gavage to the taurine group, 2 mL of AGE at a dose of 100 mg/kg/day [8] to the AGE group, and saline suspension to the control group were given as a nutritional supplement for the same duration, volume, and route.

**Figure 1:**
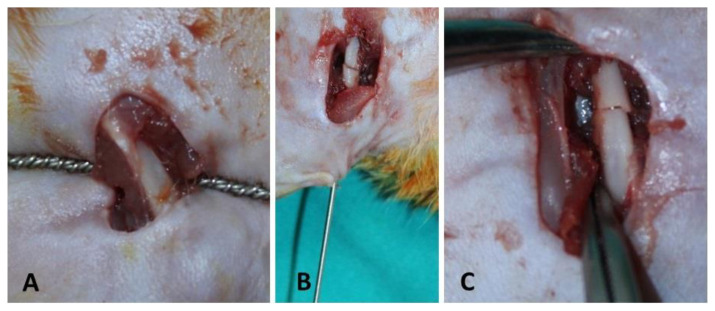
Creation of osteotomy with Gigli wire at the middiaphyseal region of rat femur (A), K-wire stabilization of the mini-open osteotomy line (B), and the appearance of rat femur osteotomy line (C).

A middiaphyseal transverse osteotomy was performed (by G Koca and M Mısırlıoğlu) with the help of a mini-open right femur lateral longitudinal incision (Figure 1A). Then, 1.6 mm Kirschner wires fixing the standard 1 mm osteotomy line were cut from the outer surface of the knee joint to prevent movement limitation (Figures 1B and 1C.) [22]. Due to surgical interventions performed in hygienic conditions and postoperative antibiotic therapy, no significant infection findings were found in physical examination, clinical findings, and histological studies. Six weeks after the osteotomy, the femoral and whole-body scintigraphies of all rats were evaluated under general anesthesia. Then, blood samples obtained through heart puncture were taken for biochemical analysis. Afterward, the rats were sacrificed under general anesthesia, and all the right femurs were removed and preserved in saline-soaked gauze bandages, at −20 °C until a microcomputed-tomography (micro-CT) scan and the histological analysis.

### 2.3. Scintigraphic evaluation

The osteoblastic activity was evaluated by hydroxymethylene diphosphonate (HDP, Eczacıbaşı-Monrol, Turkey) bounded with technetium-99m (Tc99m) injected through the tail vein under general anesthesia. Dynamic perfusion and blood pool phase images were obtained under the gamma camera (Siemens E. Cam Siemens Medical Solutions, Hoffman Estates, IL, USA), immediately after injection of 74 MBq (2mCi), HDP radiolabeled Tc99m, and whole-body scans and late-phase bone images were obtained 2 h later. The increased uptake of bone-specific radiotracer suggests that enhanced fracture healing has been recorded as increased osteoblastic activity [23]. Afterward, soft tissues were stripped from the bone to eliminate the background osteoblastic activity.

**Figure 2:**
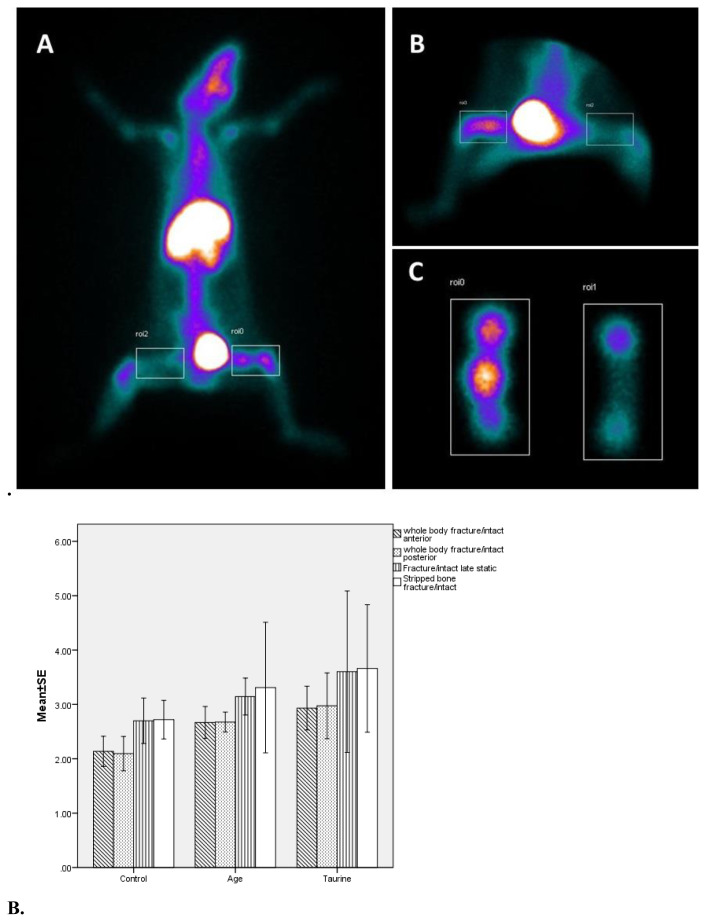
**A.** Scintigraphic images of the region of interest (ROI) drawings from the whole-body static posterior (A), static anterior (B), and stripped osteoporotic rat femur (C). **B.** Graphical comparison of scintigraphic parameters of osteoporotic rat groups’ WB F/I ROI anterior: whole-body fracture/intact ROI, WB F/I ROI posterior, F/I late static, stripped bone F/I.

Scintigraphic quantitative measurements were made by drawing regions of interest (ROI) in equal pixels, including both fractured femurs and the contralateral intact side. Radioactivity counts reflecting osteoblastic activity in ROIs were obtained from the images by quantitative bone scintigraphy analyses (Figures 2A and 2B). The whole-body osteoblastic activity values were obtained from the proportion of the ROI fractured zone counts to the intact one.

### 2.4. Biochemical evaluation

The Eppendorf tubes with 2 cc blood samples were centrifuged at 3000–3500 rpm to obtain serum and stored at −80 °C until analysis for the biochemical parameters. The serum concentrations level of alkaline phosphatase (ALP), calcium, and phosphate, which are indices of bone formation, were measured with a Roche Cobas 8000 autoanalyzer (Roche Diagnostics, USA). Osteocalcin levels (pg/mL) were determined using ELISA kits (Cusabio Biotech Co. USA).

### 2.5. Histologic evaluation

The isolated right femurs were fixed with 10% formaldehyde, decalcified, sectioned into 5 μm, stained, and photographed for histological studies. The new bones were evaluated objectively by measuring the diameter and volume of the callus. The parameters for histological analyzes such as cortex morphology, inflammation, osteoblast number, and periosteum morphology were evaluated by numerical grading as described previously by Santić et al. [24].

### 2.6. Microcomputed-tomographic evaluation

**Figure 3:**
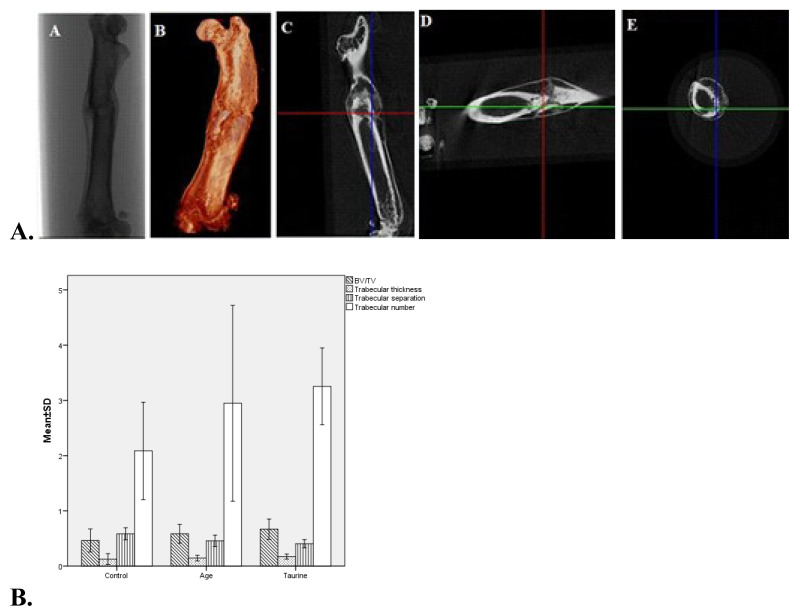
**A.** Histomorphological (micro-CT) two-dimensional (A), three-dimensional (B), coronal (C), sagittal (D), and transaxial (E) images of the rat’s right femur osteoporotic fracture line at the taurine group (T6). **B.** Graphical comparison of micro-CT parameters of osteoporotic rat groups’ BV/TV (p = 0.03): bone volume/total volume, TbTh (p > 0.05): trabecular thickness, TbSp (p <0.01): trabecular separation, TbN (p < 0.01): trabecular number.

Histomorphometric analysis of the right femurs was performed using high-resolution micro-CT (SkyScan 1174; Bruker-micro-CT, Kontich, Belgium; at 50 kV, 0.25 mm filter with 33 μm pixels) 800 μA, 50 kVp, and 21 μm projections within 180° rotation and 4000 ms scanning time. Standard software programs were used to perform qualitative and quantitative analyses of rat femurs geometry including bone volume/tissue volume (BV/TV, %), trabecular thickness (TbTh, μm), and trabecular separation (TbSp, μm), trabecular number (TbN, mm^−1^), bone surface density (BSD, mm^2^/^/^mm^3^). The region of interest (ROI) was standardized to be 10 mm proximal and distal from the fracture line. Three-dimensional images were reconstructed using scanner software (CTan) (version 1.20.3.0) for quantitative analysis (Figure 3A).

### 2.7. Statistical analysis

Statistical analyzes were performed using IBM SPSS for Windows (IBM Corporation, version 22.0, Armonk, NY, USA). Data distribution was examined by the Shapiro-Wilk test. Descriptive statistics were calculated as mean, standard deviation, minimum, and maximum values for normally distributed data, and median (range) for nonnormally distributed data. Groups were compared using one-way ANOVA for normally distributed data and the Kruskal-Wallis test for nonnormally one. Posthoc tests for group comparisons such as LSD, Dunnett’s t, or Dunnett’s T3 tests were used according to the homogeneity of variance. Since the chi-square test does not meet the assumptions, the number of cases (n) and ratios (%) were presented for the categorical variables. A p-value <0.05 was considered statistically significant.

## 3. Results

### 3.1. Scintigraphic measurements

The osteoblastic activity values of the taurine group in the whole-body fractured/intact bone ROI anterior and posterior scintigraphic images were significantly higher than the control group (p = 0.005, p = 0.023, respectively). The osteoblastic activity values of taurine and AGE groups were higher than the control group in late static, stripped bone, and other scintigraphic parameters; however, they were not statistically significant (Table 1, Figure 2B).

### 3.2. Biochemical measurements

**Figure 4:**
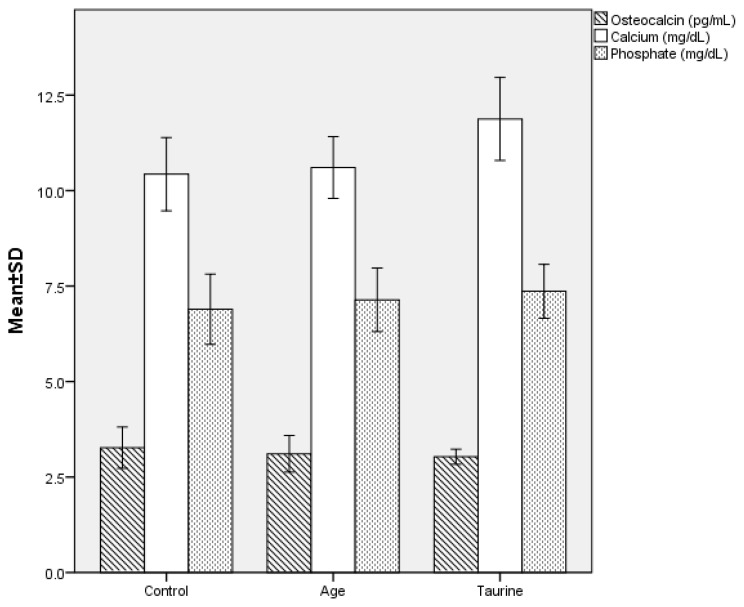
Comparison of serum biochemical parameters such as osteocalcin, calcium, (p value < 0.01), and phosphate levels of ovariectomy-induced osteoporotic rat groups 6 weeks after osteotomy.

Among the groups, only the calcium levels of the taurine group were significantly higher than the AGE and control groups respectively (p < 0.001 for both values). The osteocalcin and ALP values were lower and phosphate values were higher in the taurine and AGE groups, respectively, compared to the control group, however, these differences were not statistically significant (p > 0.05) (Figure 4). The biochemical, histological, morphometric, and scintigraphic measurements for groups are presented in Table 1.

### 3.3. Histologic measurements

**Figure 5:**
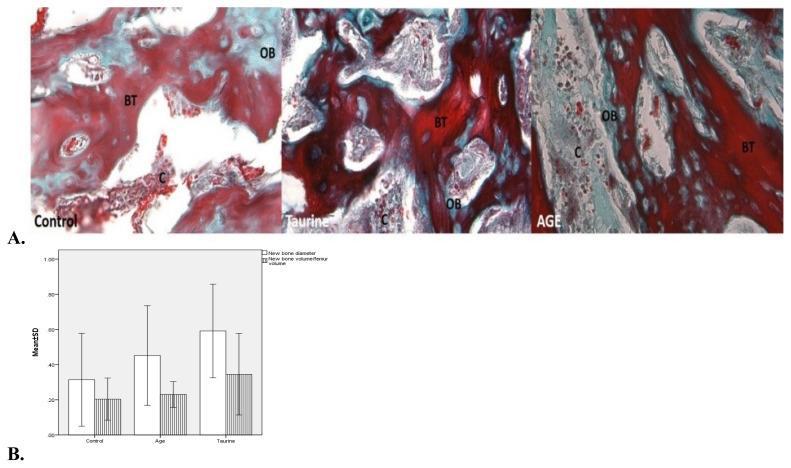
**A.** Histologically representative photomicrographs of the fracture line and new bone areas of control, taurine, and aqueous garlic extract groups; areas of fibrocartilaginous callus (C), new bone trabeculae (BT), and old bone (OB). Masson trichrome, ×400. **B.** Graphical comparison of histological parameters of the groups’ new bone diameter (p = 0.002) and new bone volume/femur volume (p = 0.032).

In histopathological examinations, larger areas of new mineralized callus formation were observed in the taurine and AGE groups, respectively, compared to the control group (Figure 5A). The taurine group also had significantly higher values for new bone diameter and new bone volume/femur volume values than the control group (p = 0.002 and p = 0.032, respectively) (Table 1, Figure 5B). While higher trabecular-compact callus scores were detected in the taurine and AGE groups, respectively, compared to the control group, osteoblast activity count scores were higher in the taurine group compared to the control group (Table 2).

### 3.4. Microcomputed-tomographic measurements

The histomorphometric analyzes showed that BV/TV and BSD values were significantly increased in the taurine and AGE groups, compared to the control (p = 0.001, p < 0.001, respectively). There was a significant difference between groups in TbN values (p = 0.003) and the difference was significant in the taurine group compared to the control (p < 0.001). Moreover, the difference was significant between groups in terms of TbSp values and the TbSp values of the taurine and AGE groups respectively were significantly lower compared to the control group (p < 0.001 for both values). In the taurine group, the BV/TV, TbN, and BSD values were the highest compared to other groups. The TbTh values of the taurine and AGE groups were higher than the control group; however, no statistically significant difference was found (p > 0.05) (Table 1, Figure 3B).

## 4. Discussion

Despite new treatment modalities and developments, OPFs are still a common and important orthopedic problem due to delayed healing, morbidity, mortality, and socioeconomic burdens [3,23]. Since taurine and AGE have proven efficacy in the treatment of osteoporosis, and patients with osteoporosis are prone to fractures, it is important to know the effects of these on the healing of OPFs. Subsequently, nutrition is important in the prevention of osteoporosis, as well as in the healing of OPFs [2,4]. However, so far, there is no comprehensive study on OPF healing with multimodal methods similar to our study. In this study, the positive effects of taurine and AGE, respectively, on the healing of OPFs were demonstrated by many diagnostic modalities.

Osteoblastic markers, ALP, and osteocalcin increase in osteoporosis when bone turnover increases, whereas their decrease indicates antiosteoporotic activity and stable bone formation [9,19]. Due to hypocalcemia in menopause, bone mineral density decreases, and the risk of fracture increases. It has been shown that taurine has a positive effect on bone mineral density and content and increases serum Ca and phosphate levels in ovariectomy-induced osteoporotic rats [13]. Taurine supplementation has been shown to significantly reduce osteocalcin levels in B12-deficient infant rats [9]. The reason why taurine could not show positive effects on osteoporotic bones in ovariectomized rats fed a calcium-poor diet was explained as the close relationship between calcium and protein activities in bone metabolism with each other [13]. Regarding that, we fed the rats a standard diet containing calcium. Studies have shown that garlic nutritional supplements decrease the hypogonadal bone loss caused by ovariectomy and improve osteoporotic low bone densities. Additionally, AGE reduces calcium and phosphate excretion from urine, while it decreases serum ALP and osteocalcin levels in ovariectomized rats [2,7]. It has been reported that AGE as a dietary supplement improves the biomechanical properties of bone, reduces fragility, and increases serum calcium levels in senile osteoporotic elderly male rats [25]. Similarly, in our study, while the control group had high ALP and osteocalcin values, and low calcium and phosphate values consistent with osteoporosis, taurine, and AGE were shown to have effective antiosteoporotic effects in the improvement of OPF with biochemical parameters, respectively. Compared to other groups, the lowest ALP, and osteocalcin levels and the highest calcium, and phosphate levels in the taurine group indicate that taurine may have the highest healing effect on OPFs.

A study examining the histological analyzes of the vertebrae and long bones of postweaning rats have found that oral taurine supplementation increases bone mass, and prevents osteoporosis and growth retardation by normalizing growth hormone in hepatocytes and activating osteoblasts [9]. In another study evaluating the histological effects of AGE on rat bones, an increase in bone marrow cellularity and myeloid-erythroid cells was observed [21]. Moreover, analysis of in vivo data of micro-CT and histological analyzes on rat calvarial bones showed us that, garlic is an effective agent in preventing severe osteoporosis-related bone loss and osteolysis [18]. In our histologic analysis, we found that taurine and AGE are effective in OPF healing, respectively, with large mineralized trabecular-compact callus areas and osteoblastic activity. Moreover, unlike previous osteoporosis studies, we have examined both quantitative and qualitative histological data and we found that the taurine group has shown significantly higher values for new bone diameter and new bone volume/femur volume values than the control group. Higher trabecular-compact callus scores were detected in both taurine and AGE groups compared to the control group, and also osteoblast activity count scores were higher in the taurine group compared to the control group.

Experimental studies on ovariectomy-induced osteoporotic animals have shown that BV/TV, TbN, TbTh, and BSD values decrease and trabecular bone weakens, while TbSp increases by micro-CT [3,4,18]. Providing taurine as a nutritional supplement in rats prevents osteoporotic bone loss after ovariectomy and increases bone volume, TbTh, and TbN values (13). Similarly, it was reported that growth plate thickness, BV/TV, TbTh, and TbN parameters increased and osteoporosis improved in rats fed taurine-rich Oysters [26]. In another study, it was determined that oral AGE decreased BV/TV and osteoclast number in osteoporotic rats [18]. In a metaanalysis study, high TbN, BV/TV ratio, and low TbSp values were reported in osteoporotic rats fed a phytoestrogen-supplemented diet [19]. Likewise, we have evaluated the bone microarchitecture and the efficacy of treatment in OPFs more precisely by quantitative micro-CT analyses of cortical and trabecular bone. Consistent with the literature, our study showed higher osteoporosis parameters in the control group, higher BV/TV, BSD, TbTh, and TbN values, and lower TbSp values in the taurine and AGE groups, respectively.

Scintigraphic imaging provides information about bone metabolism, blood flow, and bone turnover in direct proportion to osteoblastic activity, and also it is a sensitive and useful method in the evaluation of OPF healing in experimental rat models [27]. In an experimental study investigating the effects of different implants on the healing of bone fractures, an increase in osteoblastic activity of cortical and trabecular rat bone scintigraphy was shown to be a finding consistent with fracture healing [24]. Similarly, in our study, we observed that the taurine group had the highest osteoblastic activity, implementing the best fracture healing by histomorphologic methods.

Our limitations are the low number of animals in the groups due to ethical reasons, and no groups with different durations and doses were included after osteotomy. The importance of our study is that the improvement in the OPF has been supported by multimodal quantitative assessments including biochemical, histological, μCT, and scintigraphic parameters. As our study was the preliminary study for the effects of taurine and aqueous garlic extract on osteoporotic fracture healing, further large-scale, randomized, controlled molecular, experimental, and clinical studies, taking into account many heterogeneities, including dosage, duration, form, components, and route of administration, may be required.

## 5. Conclusion

Our findings suggest that taurine and AGE are promising agents for treating postmenopausal hip fractures, that deserve comprehensive, controlled clinical studies on OPFs. To the best of our knowledge, as a first, we have demonstrated that both taurine and AGE are oral nutritional supplements that improve OPF healing detected by multimodal quantitative evaluations including biochemical, histological, micro-CT, and scintigraphic parameters.

## Figures and Tables

**Table 1:** Biochemical, histological, microtomography, and scintigraphic evaluation of taurine, aqueous garlic extract, and control groups. Each value is presented as mean ± SD (min-max) or median (range).

Groups	Control (C) (n = 8)	Taurine (n = 8)	Aqueous garlic extract (AGE) (n = 8)	*p*-Value	Pairwise comparisons *p-Value
**Biochemical evaluation**					
Osteocalcin (ng/mL)	3.27 ± 0.27 (2.92–3.65)	3.03 ± 1.00 (2.90–3.16)	3.11 ± 0.24 (2.63–3.38)	0.105	-
ALP (U/L)	181.13 ± 27.28 (141–212)	148.75 ± 14.24 (127–173)	163.88 ± 35.92 (109–214)	0.083	-
Calcium (mg/dL)	10.43 ± 0.48 (9.53–10.90)	11.88 ± 0.54 (10.97–12.69)	10.61 ± 0.40 (10.12–11.40)	<.001*	Ta-C (p < 0.001) Ta-AGE (p < 0.001)
Phosphate (mg/dL)	6.89 ± 0.46 (6.24–7.66)	7.37 ± 0.35 (6.77–7.85)	7.14 ± 0.42 (6.48–7.81)	0.096	-
**Histological evaluation**					
New bone diameter (mm)	0.24 (0.37)	0.63 (0.39)	0.43 (0.35)	0.003*¥	Ta-C (p = 0.002*)
New bone volume/Femur volume (%)	0.21 ± 0.06 (0.11–0.29)	0.35 ± 0.12 (0.20–0.54)	0.23 ± 0.04 (0.18–0.28)	0.004*Ϯ	Ta-C (p = 0.032*)
**Microcomputed-tomographic Analysis**					
Bone volume/Tissue volume (BV/TV) (%)	0.46 ± 0.11 (0.28–0.58)	0.67 ± 0.09 (0.49–0.82)	0.59 ± 0.09 (0.44–0.70)	0.001*Ϯ	Ta-C (p < 0.001) AGE-C (p = 0.03)
Trabecular thickness (TbTh) (mm)	0.13 ± 0.05 (0.07–0.22)	0.17 ± 0.02 (0.14–0.21)	0.15 ± 0.03 (0.11–0.17)	0.054Ϯ	-
Trabecular separation (TbSp) (mm)	0.59 ± 0.05 (0.50–0.65)	0.41 ± 0.04 (0.35–0.46)	0.46 ± 0.05 (0.39–0.52)	<0.001*Ϯ	Ta-C (p < 0.001) AGE-C (p < 0.001)
Trabecular number (TbN) (mm^−^^1^)	2.09 ± 0.44 (1.65–2.89)	3.25 ± 0.35 (2.79–3.69)	2.95 ± 0.89 (1.96–4.31)	0.003*Ϯ	Ta-C (p<0.001)
Bone. surface density (BSD) (g/cm^3^)	8.66 ± 0.92 (7.48–10.48)	12.10 ± 0.58 (11.44–12.98)	10.23 ± 2.26 (6.41–13.33)	<0.001*Ϯ	Ta-C (p < 0.001) AGE-C (p = 0.023)
**Scintigraphic evaluation**					
WB F/I Roi-Anterior	2.14 ± 0.39 (1.62–2.82)	2.93 ± 0.57 (2.15–3.75)	2.67 ± 0.42 (1.97–3.19)	0.008*Ϯ	Ta-C (p = 0.005)
WB F/I ROI-P	2.04 (1.26)	2.84 (2.62)	2.72 (0.87)	0.021*¥	Ta-C (p = 0.023)
F/I Posterior Late-Static	2.85 (1.91)	2.84 (6.55)	3.17 (1.47)	0.430¥	-
F/I Stripped Bone	2.57 (1.44)	2.85 (5.07)	2.87 (5.22)	0.391¥	-

Ϯ One-way ANOVA,

¥ Kruskal-Wallis test,

* p < 0.05.

In the column of paired comparisons, the values in which p values and group names are given, show statistically significant differences.

C: control, Ta: taurine, ALP: alkaline phosphatase, BV/TV: bone volume/total volume, WB F/I ROI-anterior: whole-body fracture/intact region of interest anterior.

**Table 2:** Crosstable for histomorphometric evaluation. In histological analyzes, callus and cortex morphology, inflammation, osteoblasts number, and periosteum parameters were evaluated by numeric grading as described previously (24).

Groups	Grade	Control (n = 8)	Taurine (n = 8)	AGE (n = 8)
**Callus morphology n (%)**	0	2 (0.25)	0	0
	1	3 (37.5)	1 (12.5)	2 (25.0)
	2	2 (25.0)	2 (25.0)	4 (50.0)
	3	1 (12.5)	5 (62.5)	2 (25.0)
**Cortex morphology n (%)**	0	2 (25.0)	0	0
	1	5 (62.5)	0	2 (25.0)
	2	1 (12.5)	8 (100.0)	6 (75.0)
**Inflammatory responses n (%)**	0	1 (12.05)	0	0
	1	6 (75.0)	5 (62.5)	5 (62.5)
	2	1 (12.5)	3 (37.5)	2 (25.0)
**Osteoblast count n (%)**	0	0	0	0
	1	7 (87.5)	2 (25.0)	5 (62.5)
	2	1 (12.5)	4 (50.0)	3 (37.5)
	3	0	2 (25.0)	0
**Periosteal vascularity n (%)**	0	2 (25.0)	0	0
	1	6 (75.0)	4 (75.0)	6 (75.0)
	2	0	4 (25.0)	2 (25.0)

## References

[1] Cheung WH Miclau T Chow SK Yang FF Alt V Fracture healing in osteoporotic bone Injury 2016 47 2 21 26 10.1016/S0020-1383(16)47004-X 27338222

[2] Mukherjee M Das AS Das D Mukherjee S Mitra S Mitra C Role of peritoneal macrophages and lymphocytes in the development of hypogonadal osteoporosis in an ovariectomized rat model: possible phytoestrogenic efficacy of oil extract of garlic to preserve skeletal health Phytotherapy Research 2007 21 11 1045 1054 https://doiorg/10.1002/ptr.2209 17600860 10.1002/ptr.2209

[3] Ibrahim N Mohamad S Mohamed N Shuid AN Experimental fracture protocols in assessments of potential agents for osteoporotic fracture healing using rodent models Current Drug Targets 2013 14 14 1642 1650 https://doiorg/10.2174/1389450114666131216224003 24350807 10.2174/1389450114666131216224003

[4] Choi MJ Taurine May Modulate Bone in Cholesterol Fed Estrogen Deficiency-Induced Rats Advances in Experimental Medicine and Biology 2017 975 Pt 2 1093 1102 https://doiorg/10.1007/978-94-024-1079-2_87 28849525 10.1007/978-94-024-1079-2_87

[5] Chen Y Sun J Dou C Li N Kang F Alliin Attenuated RANKL-Induced Osteoclastogenesis by Scavenging Reactive Oxygen Species through Inhibiting Nox1 International Journal of Molecular Sciences 2016 17 9 1 13 https://doiorg/10.3390/ijms17091516 10.3390/ijms17091516 PMC5037793 27657047

[6] Cheong SH Chang KJ The preventive effect of fermented milk supplement containing tomato (Lycopersicon esculentum) and taurine on bone loss in ovariectomized rats Advances in Experimental Medicine and Biology 2009 643 333 340 https://doiorg/10.1007/978-0-387-75681-3_34 19239164 10.1007/978-0-387-75681-3_34

[7] Putnam SE Scutt AM Bicknell K Priestley CM Williamson EM Natural products as alternative treatments for metabolic bone disorders and for maintenance of bone health Phytotherapy Research 2007 21 2 99 112 https://doiorg/10.1002/ptr.2030 17106868 10.1002/ptr.2030

[8] Gupta RC Win T Bittner S Taurine analogs; a new class of therapeutics: retrospect and prospects Current Medicinal Chemistry 2005 12 17 2021 2039 https://doiorg/10.2174/0929867054546582 16101502 10.2174/0929867054546582

[9] Roman-Garcia P Quiros-Gonzalez I Mottram L Lieben L Sharan K Vitamin B_12_-dependent taurine synthesis regulates growth and bone mass The Journal of Clinical Investigation 2014 124 7 2988 3002 https://doiorg/10.1172/JCI72606 24911144 10.1172/JCI72606 PMC4071367

[10] Wu G Important roles of dietary taurine, creatine, carnosine, anserine and 4-hydroxyproline in human nutrition and health Amino Acids 2020 52 3 329 360 https://doiorg/10.1007/s00726-020-02823-6 32072297 10.1007/s00726-020-02823-6 PMC7088015

[11] D’Eufemia P Finocchiaro R Celli M Raccio I Zambrano A Taurine deficiency in thalassemia major-induced osteoporosis treated with neridronate Biomedicine & Pharmacotherapy 2010 64 4 271 274 https://doiorg/10.1016/j.biopha.2009.06.014 20359847 10.1016/j.biopha.2009.06.014

[12] Choi MJ Seo JN Effect of taurine feeding on bone mineral density and bone markers in rats Advances in Experimental Medicine and Biology 2013 776 51 58 https://doiorg/10.1007/978-1-4614-6093-0_6 23392870 10.1007/978-1-4614-6093-0_6

[13] Choi MJ Chang KJ Lee JW Jung YJ Beneficial Function of Taurine on Bone Metabolism in Alcohol-Fed OVX Rat Model Advances in Experimental Medicine and Biology 2017 975 Pt 2 1059 1069 https://doiorg/10.1007/978-94-024-1079-2_84 28849522 10.1007/978-94-024-1079-2_84

[14] Van Stijn MF Bruins AA Vermeulen MA Witlox J Teerlink T Effect of oral taurine on morbidity and mortality in elderly hip fracture patients: a randomized trial International Journal of Molecular Sciences 2015 16 6 12288 12306 https://doiorg/10.3390/ijms160612288 26035756 10.3390/ijms160612288 PMC4490444

[15] Ansary J Forbes-Hernández TY Gil E Cianciosi D Zhang J Potential Health Benefit of Garlic Based on Human Intervention Studies: A Brief Overview Antioxidants (Basel) 2020 9 7 619 https://doiorg/10.3390/antiox9070619 32679751 10.3390/antiox9070619 PMC7402177

[16] Ohtani M Nishimura T The preventive and therapeutic application of garlic and other plant ingredients in the treatment of periodontal diseases Experimental and Therapeutic Medicine 2020 19 2 1507 1510 https://doiorg/10.3892/etm.2019.8382 32010331 10.3892/etm.2019.8382 PMC6966117

[17] Arreola R Quintero-Fabián S López-Roa RI Flores-Gutiérrez EO Reyes-Grajeda JP Immunomodulation and anti-inflammatory effects of garlic compounds Journal of Immunology Research 2015 2015 401630 https://doiorg/10.1155/2015/401630 25961060 10.1155/2015/401630 PMC4417560

[18] Yang J Tang R Yi J Chen Y Li X Diallyl disulfide alleviates inflammatory osteolysis by suppressing osteoclastogenesis via NF-κB-NFATc1 signal pathway FASEB Journal: Official Publication of the Federation of American Societies for Experimental Biology 2019 33 6 7261 7273 https://doiorg/10.1096/fj.201802172R 30857415 10.1096/fj.201802172R PMC6554198

[19] Fu SW Zeng GF Zong SH Zhang ZY Zou B Systematic review and meta-analysis of the bone protective effect of phytoestrogens on osteoporosis in ovariectomized rats Nutrition Research (New York) 2014 34 6 467 477 https://doiorg/10.1016/j.nutres.2014.05.003 10.1016/j.nutres.2014.05.003 25026913

[20] Ahmadian F Mozaffari-Khosravi H Azaraein MH Faraji R Zavar-Reza J The effect of consumption of garlic tablet on proteins oxidation biomarkers in postmenopausal osteoporotic women: A randomized clinical trial Electronic Physician 2017 9 11 5670 5675 https://doiorg/10.19082/5670 29403603 10.19082/5670 PMC5783112

[21] Odiase DE Osazee LO Histological Effects Aqueous Extract of Allium sativum (Alliaceae) Bulb on Bone and Spleen of Adult Wistar Rats Journal of Applied Sciences and Environmental Management 2017 21 3 538 544 https://doiorg/10.4314/jasem.v21i3.15

[22] Kuo YJ Sun JS Rau G Chen CH Tsai TH Tsuang YH Better Osteoporotic Fracture Healing with Sintered Dicalcium Pyrophosphate (SDCP) Treatment: A Rat Femoral Fracture Model The Journal of Histochemistry and Cytochemistry 2014 62 8 565 576 https://doiorg/10.1369/0022155414538264 24828625 10.1369/0022155414538264

[23] Özmeriç A Tanoğlu O Ocak M Çelik HH Fırat A Intramedullary implants coated with cubic boron nitride enhance bone fracture healing in a rat model Journal of Trace Elements in Medicine and Biology 2020 62 126599 https://doiorg/10.1016/j.jtemb.2020.126599 32629303 10.1016/j.jtemb.2020.126599

[24] Santić V Cvek SZ Sestan B Bobinac D Tudor A Treatment of tibial bone defect with rotational vascular periosteal graft in rabbits Collegium Antropologicum 2009 33 1 43 50 19408602

[25] Liu Y You M Shen J Xu Y Li L Allicin Reversed the Process of Frailty in Aging Male Fischer 344 Rats With Osteoporosis The Journal of Gerontology Series A, Biological Sciences and Medical Sciences 2020 75 5 821 825 https://doiorg/10.1093/gerona/glz205 10.1093/gerona/glz205 PMC7164536 31541608

[26] Moon PD Kim MH Lim HS Oh HA Nam SY Taurine, a major amino acid of oyster, enhances linear bone growth in a mouse model of protein malnutrition BioFactors 2015 41 3 190 197 https://doiorg/10.1002/biof.1213 25963419 10.1002/biof.1213

[27] Cheng C Alt V Pan L Thormann U Schnettler R Preliminary evaluation of different biomaterials for defect healing in an experimental osteoporotic rat model with dynamic PET-CT (dPET-CT) using F-18-sodium fluoride (NaF) Injury 2014 45 3 501 505 https://doiorg/10.1016/j.injury.2013.11.023 24332163 10.1016/j.injury.2013.11.023

